# 4-(1-Naphth­yl)benzonitrile

**DOI:** 10.1107/S1600536810042108

**Published:** 2010-11-24

**Authors:** Carlos F. Lima, Ligia R. Gomes, Luís M. N. B. F. Santos, John Nicolson Low

**Affiliations:** a Centro de Investigação em Química, Departamento de Química e Bioquímica, Faculdade de Ciências, Universidade do Porto, Rua do Campo Alegre, 687, P-4169 007 Porto, Portugal; b REQUIMTE, Departamento de Química e Bioquímica, Faculdade de Ciências, Universidade do Porto, Rua do Campo Alegre, 687, P-4169 007 Porto, Portugal; c Department of Chemistry, University of Aberdeen, Meston Walk, Old Aberdeen AB24 3UE, Scotland

## Abstract

The title compound, C_17_H_11_N, crystallizes with two mol­ecules in the asymmetric unit which are linked by a weak C—H⋯N hydrogen bond. The dihedral angles between the benzene ring and the naphthalene ring system in the two mol­ecules are 60.28 (3) and 60.79 (3)°. In the crystal, mol­ecules are linked into a three-dimensional network by weak C—H⋯π inter­actions.

## Related literature

For the structure of the related compound 1-(3,4,5-trimeth­oxy­phen­yl)naphthalene, see: Suthar *et al.* (2005 ▶).
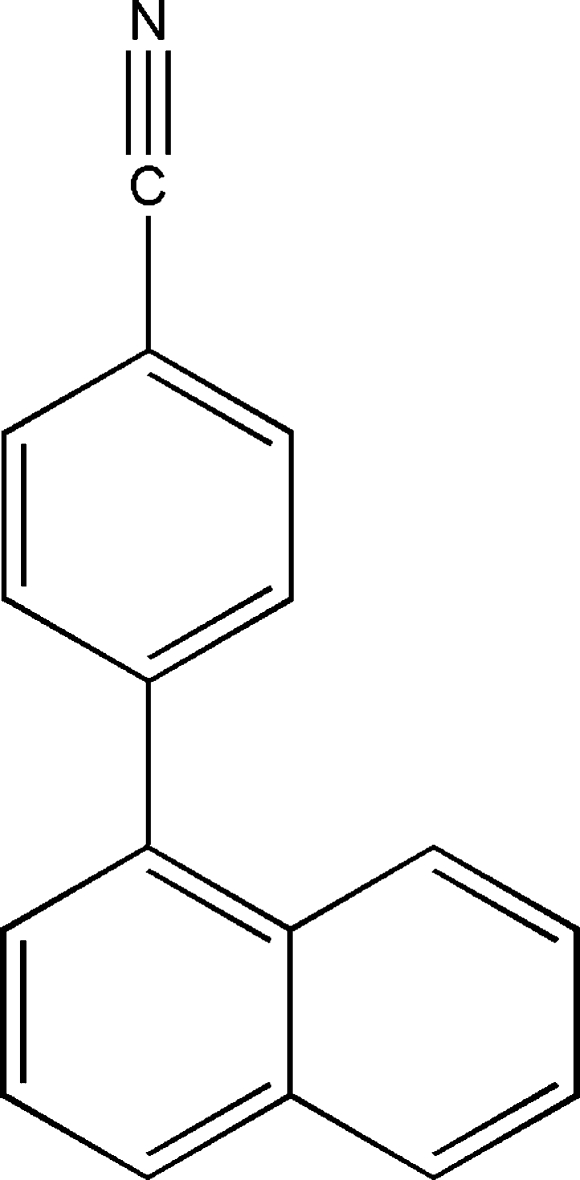


## Experimental

### 

#### Crystal data

C_17_H_11_N
*M* *_r_* = 229.27
Triclinic, 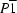

*a* = 7.3387 (3) Å
*b* = 11.3461 (5) Å
*c* = 15.5804 (7) Å
α = 71.237 (2)°
β = 89.981 (2)°
γ = 87.647 (2)°
*V* = 1227.22 (9) Å^3^
*Z* = 4
Mo *K*α radiation
μ = 0.07 mm^−1^
*T* = 150 K
0.40 × 0.18 × 0.06 mm

#### Data collection

Bruker SMART APEX diffractometer
Absorption correction: multi-scan (*SADABS*; Bruker, 2004 ▶) *T*_min_ = 0.972, *T*_max_ = 0.996
17670 measured reflections
7450 independent reflections
5228 reflections with *I* > 2σ(*I*)
*R*_int_ = 0.029

#### Refinement

*R*[*F* ^2^ > 2σ(*F* ^2^)] = 0.049
*wR*(*F* ^2^) = 0.135
*S* = 1.02
7450 reflections
325 parameters
H-atom parameters constrained
Δρ_max_ = 0.29 e Å^−3^
Δρ_min_ = −0.26 e Å^−3^

### 

Data collection: *APEX2* (Bruker, 2004 ▶); cell refinement: *APEX2* and *SAINT* (Bruker, 2004 ▶); data reduction: *SAINT*; program(s) used to solve structure: *SHELXS97* (Sheldrick, 2008 ▶); program(s) used to refine structure: *SHELXL97* (Sheldrick, 2008 ▶) and *OSCAIL* (McArdle *et al.*, 2004 ▶); molecular graphics: *PLATON* (Spek, 2009 ▶); software used to prepare material for publication: *SHELXL97*.

## Supplementary Material

Crystal structure: contains datablocks global, I. DOI: 10.1107/S1600536810042108/fl2322sup1.cif

Structure factors: contains datablocks I. DOI: 10.1107/S1600536810042108/fl2322Isup2.hkl

Additional supplementary materials:  crystallographic information; 3D view; checkCIF report

## Figures and Tables

**Table 1:** Hydrogen-bond geometry (Å, °) *Cg*1, *Cg*3, *Cg*5, *Cg*6 and *Cg*7 are the centroids of the C11–C110, C111–C116, C21–C210, C28–C210 and C211–C216 rings, respectively.

*D*—H⋯*A*	*D*—H	H⋯*A*	*D*⋯*A*	*D*—H⋯*A*
C113—H113⋯N42	0.95	2.58	3.4804 (17)	158
C14—H14⋯*Cg*6^i^	0.95	2.99	3.9165 (14)	165
C15—H15⋯*Cg*5^i^	0.95	2.51	3.4128 (14)	160
C17—H17⋯*Cg*3^ii^	0.95	2.86	3.6648 (15)	144
C25—H25⋯*Cg*1^iii^	0.95	2.52	3.4155 (14)	158
C27—H27⋯*Cg*7^iv^	0.95	2.91	3.7205 (15)	144
C115—H115⋯*Cg*1^v^	0.95	2.81	3.5935 (13)	141
C215—H215⋯*Cg*5^v^	0.95	2.77	3.5740 (13)	143

Symmetry codes: (i) 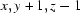; (ii) 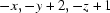; (iii) 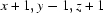; (iv) 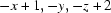; (v) 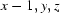.

**Fig. 1.**
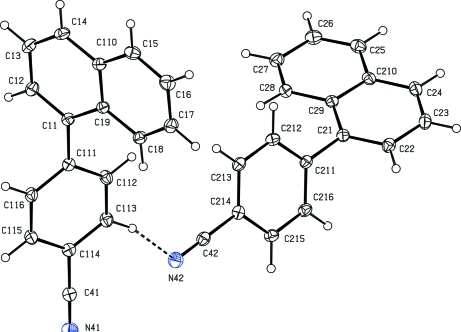
A view of (1) with our numbering scheme. Displacement ellipsoids are drawn at the 30% probability level.

**Fig. 2.**
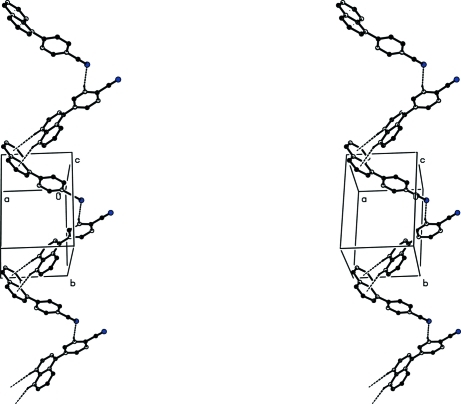
Stereoview of the chain running parallel to [10–1] formed by C—H···π dimers linked by the C—H···N hydrogen bond. Hydrogen atoms not involved in the motifs are not included.

**Fig. 3.**
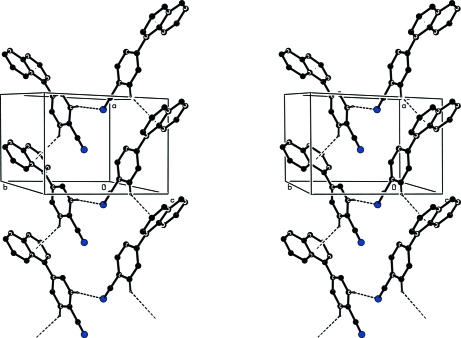
Stereoview of the ladder formed by C—H···π chains linking molecules 1 and 2. These chains are linked by the C—H···N hydrogen bond. The ladder runs parallel to the a axis. Hydrogen atoms not involved in the motifs are not included.

**Fig. 4.**
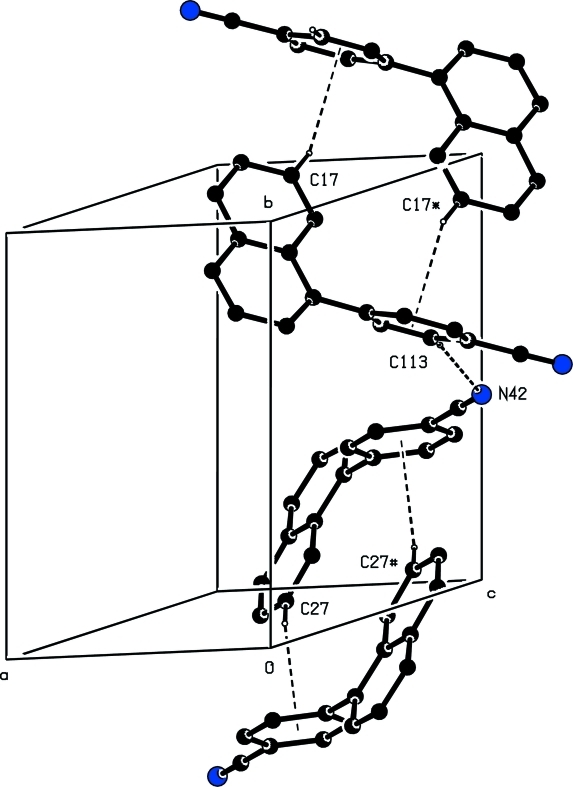
View of part of a chain of linked centrosymmetric C—H···π dimers of molecule 1 and of molecule 2. These dimers are linked by the C—H···N hydrogen bond and the chain runs parallel to the b axis. The molecule labelled *is at (-x,2 - y,1 - z) and that labelled # is at (1 - x,y,2 - z). Hydrogen atoms not involved in the motifs are not included.

**Fig. 5.**
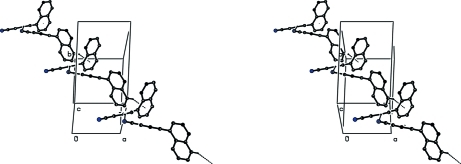
Stereoview of the chain parallel to [1–11] made up of an alternating C—H···π interaction and the C—H···N hydrogen bond. Hydrogen atoms not involved in the motifs are not included.

## supplementary crystallographic information

### Comment

The title compound crystallizes with two molecules on the asymmetric unit. The asymmetric unit was selected so that the 2 molecules were connected by the weak C113—H113···N42 hydrogen bond with H···N, 2.58 Å, C···N, 3.4804 (17)Å and the angle at H, 158°, Fig.1. Molecule 1 contains atoms C1X and molecule 2 contains atoms C2X. The two molecules show no unusual bonds or angles. The naphthalene rings form dihedral angles of 60.28 (3)° and 60.79 (3)° with the the phenyl rings for molecules 1 and 2 respectively. In the related compound 1-(3,4,5-trimethoxyphenyl)naphthalene, Suthar *et al.*, (2005), which has only one molecule in the asymmetric unit, this dihedral angle is 68.19 (10)°.

Apart from the C—H···N hydrogen bond, seven weak C—H···π interactions stabilize the supramolecular structure linking the molecules into a three dimensional network (Table 1). There are no π···π interactions

Atoms C14 and C15 form weak C—H···π interactions with the C28—C210(*Cg*6) and C21—C210(*Cg*5) naphthalene rings at (*x*,1 + *y*,-1 + *z*), forming a dimer. These dimers are linked by the C—H···N hydrogen bond to form a chain which runs parallel to [10–1], Fig. 2.

Atoms C115 and C215 form weak C—H···π interactions with the C11—C110(*Cg*1) and C21—C210(*Cg*5) naphthalene rings at (-1 + *x*,*y*,*z*) respectively, forming a ladder which runs along the *a* axis with the C—H···N hydrogen bond forming the rungs, Fig.3.

Atom C17 forms a weak C—H···π interaction with the phenyl ring C111—C116(*Cg*3) at (-*x*,2 - *y*,1 - *z*) to give a centrosymmetric dimer. Similarly, C27 forms a centrosymmetric dimer through a C—H···π with the phenyl ring C211—C216(*Cg*7). These dimers are linked together *via* the C—H···N hydrogen bond forming a chain which runs parallel to the *b* axis, Fig 4.

Atom C25 forms a weak C—H···π interaction with the C11—C110(*Cg*1) phenyl ring at (1 + *x*,1 - *y*,1 + *z*) alternating with the C—H···N hydrogen bond to form a chain which runs parallel to [1–11], Fig. 5.

The related compound 1-(3,4,5-trimethoxyphenyl)naphthalene, Suthar *et al.*, (2005) by contrast is stabilized by one C—H···π interaction. There are no π···π or C—H···O hydrogen bonds.

### Experimental

A solution of K_2_CO_3_ (6.2 mmol) in 20 ml of water was added to a solution of 1-bromonaphthalene (3.1 mmol), 4-cyanophenylboronic acid (4.5 mmol) and Pd(OAc)_2_ (1 mol %) in 20 ml of DMF. The resultant mixture was heated at 100°C for 8 h under stirring. The final solution was allowed to cool to room temperature, and extracted with ethyl acetate. The organic layer was washed with water and aqueous 0.1 *M* NaOH, dried over anhydrous sodium sulfate and evaporated. The brown solid obtained (0.45 g, 58%) was recrystallized from MeOH and sublimed under reduced pressure to yield white crystals of the title compound; ^1^H-NMR (400 MHz, CDCl_3_, 300 K, TMS) 7.98–7.92 (2*H*, m, 2-H + 8-H), 7.81 (2*H*, d, 2-H, J=8.2), 7.84–7.78 (1*H*, m), 7.64 (2*H*, d, 3-H, J=8.2), 7.61–7.40 (4*H*, m); ^13^C-NMR (100.6 MHz, CDCl_3_, 300 K) 146.6, 139.1, 134.7, 133.0, 131.9, 131.7, 129.7, 129.5, 127.9,127.6, 127.1, 126.3, 126.1, 119.8, 112.1.

### Refinement

Molecule (1) crystallized in the triclinc system; space group P-1. H atoms were treated as riding atoms with C—H(aromatic), 0.95 Å, with *U*_iso_ = 1.2Ueq(C). The positions of the H atoms were calculated and checked against a difference map during the refinement.

### Crystal data

**Table:**

C_17_H_11_N	*Z* = 4
*M**_r_* = 229.27	*F*(000) = 480
Triclinic, *P*1	*D*_x_ = 1.241 Mg m^−^^3^
*a* = 7.3387 (3) Å	Mo *K*α radiation, λ = 0.71073 Å
*b* = 11.3461 (5) Å	Cell parameters from 414 reflections
*c* = 15.5804 (7) Å	θ = 1.4–29.9°
α = 71.237 (2)°	µ = 0.07 mm^−^^1^
β = 89.981 (2)°	*T* = 150 K
γ = 87.647 (2)°	Plate, white
*V* = 1227.22 (9) Å^3^	0.40 × 0.18 × 0.06 mm

### Data collection

**Table:**

Bruker SMART APEX diffractometer	7450 independent reflections
Radiation source: fine-focus sealed tube	5228 reflections with *I* > 2σ(*I*)
graphite	*R*_int_ = 0.029
Detector resolution: 8.33 pixels mm^-1^	θ_max_ = 30.6°, θ_min_ = 1.4°
ω scans	*h* = −10→8
Absorption correction: multi-scan (*SADABS*; Bruker, 2004)	*k* = −16→16
*T*_min_ = 0.972, *T*_max_ = 0.996	*l* = −22→22
17670 measured reflections	

### Refinement

**Table:**

Refinement on *F*^2^	Primary atom site location: structure-invariant direct methods
Least-squares matrix: full	Secondary atom site location: difference Fourier map
*R*[*F*^2^ > 2σ(*F*^2^)] = 0.049	Hydrogen site location: inferred from neighbouring sites
*wR*(*F*^2^) = 0.135	H-atom parameters constrained
*S* = 1.02	*w* = 1/[σ^2^(*F*_o_^2^) + (0.0657*P*)^2^ + 0.1709*P*] where *P* = (*F*_o_^2^ + 2*F*_c_^2^)/3
7450 reflections	(Δ/σ)_max_ = 0.001
325 parameters	Δρ_max_ = 0.29 e Å^−^^3^
0 restraints	Δρ_min_ = −0.26 e Å^−^^3^

### Special details

**Table:**

Geometry. All e.s.d.'s (except the e.s.d. in the dihedral angle between two l.s. planes) are estimated using the full covariance matrix. The cell e.s.d.'s are taken into account individually in the estimation of e.s.d.'s in distances, angles and torsion angles; correlations between e.s.d.'s in cell parameters are only used when they are defined by crystal symmetry. An approximate (isotropic) treatment of cell e.s.d.'s is used for estimating e.s.d.'s involving l.s. planes.
Refinement. Refinement of *F*^2^ against ALL reflections. The weighted *R*-factor *wR* and goodness of fit *S* are based on *F*^2^, conventional *R*-factors *R* are based on *F*, with *F* set to zero for negative *F*^2^. The threshold expression of *F*^2^ > σ(*F*^2^) is used only for calculating *R*-factors(gt) *etc*. and is not relevant to the choice of reflections for refinement. *R*-factors based on *F*^2^ are statistically about twice as large as those based on *F*, and *R*- factors based on ALL data will be even larger.

### Fractional atomic coordinates and isotropic or equivalent isotropic displacement parameters (Å^2^)

**Table:**

	*x*	*y*	*z*	*U*_iso_*/*U*_eq_	
C11	0.14757 (16)	0.76418 (10)	0.38598 (8)	0.0221 (2)	
C12	0.19777 (17)	0.70862 (11)	0.32255 (8)	0.0269 (3)	
H12	0.1340	0.6389	0.3194	0.032*	
C13	0.34105 (18)	0.75237 (12)	0.26217 (8)	0.0299 (3)	
H13	0.3747	0.7109	0.2200	0.036*	
C14	0.43179 (17)	0.85400 (12)	0.26389 (8)	0.0286 (3)	
H14	0.5284	0.8831	0.2229	0.034*	
C15	0.47043 (18)	1.02562 (12)	0.32668 (9)	0.0309 (3)	
H15	0.5666	1.0554	0.2856	0.037*	
C16	0.41930 (19)	1.08849 (13)	0.38456 (10)	0.0336 (3)	
H16	0.4788	1.1619	0.3832	0.040*	
C17	0.27844 (18)	1.04476 (12)	0.44638 (9)	0.0310 (3)	
H17	0.2432	1.0889	0.4866	0.037*	
C18	0.19164 (17)	0.93904 (11)	0.44906 (8)	0.0255 (3)	
H18	0.0974	0.9104	0.4916	0.031*	
C19	0.24048 (15)	0.87157 (10)	0.38918 (8)	0.0217 (2)	
C110	0.38284 (16)	0.91668 (11)	0.32656 (8)	0.0241 (2)	
C111	−0.00642 (16)	0.71497 (10)	0.44700 (8)	0.0222 (2)	
C112	0.01541 (17)	0.67331 (11)	0.54144 (8)	0.0261 (3)	
H112	0.1317	0.6760	0.5674	0.031*	
C113	−0.12902 (17)	0.62850 (11)	0.59737 (8)	0.0269 (3)	
H113	−0.1125	0.6014	0.6613	0.032*	
C114	−0.29969 (16)	0.62316 (11)	0.55959 (8)	0.0240 (2)	
C115	−0.32370 (17)	0.66236 (12)	0.46573 (8)	0.0274 (3)	
H115	−0.4395	0.6579	0.4399	0.033*	
C116	−0.17772 (17)	0.70781 (12)	0.41035 (8)	0.0269 (3)	
H116	−0.1943	0.7346	0.3464	0.032*	
C41	−0.45145 (17)	0.57807 (11)	0.61786 (8)	0.0266 (3)	
N41	−0.57332 (16)	0.54308 (11)	0.66387 (8)	0.0347 (3)	
C21	0.60833 (16)	0.24602 (11)	1.10821 (8)	0.0226 (2)	
C22	0.64704 (17)	0.30616 (11)	1.16966 (8)	0.0271 (3)	
H22	0.5713	0.3754	1.1711	0.032*	
C23	0.79668 (18)	0.26710 (12)	1.23036 (9)	0.0302 (3)	
H23	0.8229	0.3116	1.2708	0.036*	
C24	0.90385 (17)	0.16564 (12)	1.23120 (8)	0.0291 (3)	
H24	1.0043	0.1398	1.2724	0.035*	
C25	0.97139 (17)	−0.01063 (12)	1.17380 (9)	0.0308 (3)	
H25	1.0713	−0.0375	1.2152	0.037*	
C26	0.93146 (18)	−0.07747 (12)	1.11799 (9)	0.0333 (3)	
H26	1.0023	−0.1508	1.1213	0.040*	
C27	0.78535 (18)	−0.03792 (12)	1.05543 (9)	0.0299 (3)	
H27	0.7582	−0.0848	1.0167	0.036*	
C28	0.68220 (16)	0.06749 (11)	1.05006 (8)	0.0251 (2)	
H28	0.5848	0.0934	1.0071	0.030*	
C29	0.71839 (16)	0.13901 (11)	1.10776 (8)	0.0221 (2)	
C210	0.86654 (16)	0.09829 (11)	1.17107 (8)	0.0245 (2)	
C211	0.44769 (16)	0.29091 (10)	1.04619 (8)	0.0221 (2)	
C212	0.46649 (17)	0.33005 (11)	0.95198 (8)	0.0256 (2)	
H212	0.5839	0.3271	0.9268	0.031*	
C213	0.31684 (17)	0.37292 (11)	0.89494 (8)	0.0255 (2)	
H213	0.3310	0.3987	0.8310	0.031*	
C214	0.14495 (17)	0.37801 (10)	0.93206 (8)	0.0234 (2)	
C215	0.12375 (17)	0.34016 (12)	1.02594 (8)	0.0264 (3)	
H215	0.0066	0.3440	1.0511	0.032*	
C216	0.27502 (17)	0.29704 (11)	1.08210 (8)	0.0262 (3)	
H216	0.2608	0.2713	1.1460	0.031*	
C42	−0.01204 (17)	0.42267 (11)	0.87356 (8)	0.0260 (3)	
N42	−0.13766 (16)	0.45805 (11)	0.82711 (8)	0.0352 (3)	

### Atomic displacement parameters (Å^2^)

**Table:**

	*U*^11^	*U*^22^	*U*^33^	*U*^12^	*U*^13^	*U*^23^
C11	0.0239 (5)	0.0214 (5)	0.0193 (5)	0.0010 (4)	0.0004 (4)	−0.0047 (4)
C12	0.0319 (6)	0.0242 (6)	0.0249 (6)	0.0016 (5)	0.0000 (5)	−0.0087 (5)
C13	0.0358 (7)	0.0313 (7)	0.0235 (6)	0.0072 (5)	0.0024 (5)	−0.0112 (5)
C14	0.0270 (6)	0.0335 (7)	0.0221 (6)	0.0040 (5)	0.0053 (5)	−0.0050 (5)
C15	0.0267 (6)	0.0303 (7)	0.0320 (7)	−0.0046 (5)	0.0050 (5)	−0.0047 (5)
C16	0.0328 (7)	0.0277 (7)	0.0409 (8)	−0.0076 (5)	0.0030 (6)	−0.0110 (6)
C17	0.0326 (7)	0.0295 (7)	0.0352 (7)	−0.0030 (5)	0.0030 (6)	−0.0161 (6)
C18	0.0253 (6)	0.0263 (6)	0.0257 (6)	−0.0025 (5)	0.0038 (5)	−0.0091 (5)
C19	0.0212 (5)	0.0224 (5)	0.0201 (5)	0.0016 (4)	−0.0004 (4)	−0.0053 (4)
C110	0.0221 (5)	0.0257 (6)	0.0217 (6)	0.0014 (4)	0.0009 (4)	−0.0041 (5)
C111	0.0253 (6)	0.0193 (5)	0.0215 (5)	−0.0015 (4)	0.0009 (4)	−0.0057 (4)
C112	0.0257 (6)	0.0282 (6)	0.0220 (6)	−0.0038 (5)	−0.0032 (5)	−0.0042 (5)
C113	0.0304 (6)	0.0274 (6)	0.0200 (6)	−0.0032 (5)	−0.0016 (5)	−0.0035 (5)
C114	0.0261 (6)	0.0201 (5)	0.0250 (6)	−0.0026 (4)	0.0024 (5)	−0.0059 (5)
C115	0.0252 (6)	0.0303 (6)	0.0268 (6)	−0.0033 (5)	−0.0030 (5)	−0.0089 (5)
C116	0.0292 (6)	0.0305 (6)	0.0204 (6)	−0.0017 (5)	−0.0029 (5)	−0.0073 (5)
C41	0.0280 (6)	0.0247 (6)	0.0261 (6)	−0.0014 (5)	−0.0005 (5)	−0.0067 (5)
N41	0.0327 (6)	0.0354 (6)	0.0344 (6)	−0.0034 (5)	0.0042 (5)	−0.0089 (5)
C21	0.0239 (5)	0.0225 (6)	0.0202 (5)	−0.0044 (4)	0.0023 (4)	−0.0050 (4)
C22	0.0316 (6)	0.0248 (6)	0.0260 (6)	−0.0052 (5)	0.0025 (5)	−0.0094 (5)
C23	0.0350 (7)	0.0338 (7)	0.0245 (6)	−0.0108 (5)	0.0014 (5)	−0.0122 (5)
C24	0.0268 (6)	0.0353 (7)	0.0235 (6)	−0.0074 (5)	−0.0025 (5)	−0.0064 (5)
C25	0.0255 (6)	0.0322 (7)	0.0299 (7)	0.0016 (5)	−0.0014 (5)	−0.0038 (5)
C26	0.0317 (7)	0.0291 (7)	0.0375 (7)	0.0058 (5)	0.0014 (6)	−0.0092 (6)
C27	0.0307 (6)	0.0289 (6)	0.0323 (7)	−0.0004 (5)	0.0022 (5)	−0.0130 (5)
C28	0.0242 (6)	0.0266 (6)	0.0252 (6)	−0.0022 (5)	0.0005 (5)	−0.0092 (5)
C29	0.0217 (5)	0.0232 (6)	0.0199 (5)	−0.0038 (4)	0.0024 (4)	−0.0047 (4)
C210	0.0220 (5)	0.0272 (6)	0.0217 (6)	−0.0053 (5)	0.0019 (5)	−0.0034 (5)
C211	0.0253 (6)	0.0199 (5)	0.0215 (6)	−0.0019 (4)	0.0019 (4)	−0.0071 (4)
C212	0.0251 (6)	0.0271 (6)	0.0235 (6)	−0.0019 (5)	0.0053 (5)	−0.0066 (5)
C213	0.0305 (6)	0.0252 (6)	0.0195 (5)	−0.0021 (5)	0.0034 (5)	−0.0055 (5)
C214	0.0280 (6)	0.0193 (5)	0.0221 (6)	−0.0017 (4)	−0.0002 (5)	−0.0056 (4)
C215	0.0244 (6)	0.0296 (6)	0.0241 (6)	−0.0003 (5)	0.0050 (5)	−0.0074 (5)
C216	0.0295 (6)	0.0288 (6)	0.0188 (5)	−0.0001 (5)	0.0034 (5)	−0.0057 (5)
C42	0.0309 (6)	0.0250 (6)	0.0215 (6)	−0.0028 (5)	0.0030 (5)	−0.0063 (5)
N42	0.0352 (6)	0.0409 (7)	0.0275 (6)	−0.0007 (5)	−0.0019 (5)	−0.0085 (5)

### Geometric parameters (Å, °)

**Table:**

C11—C12	1.3753 (16)	C21—C22	1.3782 (16)
C11—C19	1.4351 (16)	C21—C29	1.4323 (16)
C11—C111	1.4840 (17)	C21—C211	1.4892 (15)
C12—C13	1.4067 (18)	C22—C23	1.4099 (17)
C12—H12	0.9500	C22—H22	0.9500
C13—C14	1.3627 (19)	C23—C24	1.3638 (18)
C13—H13	0.9500	C23—H23	0.9500
C14—C110	1.4190 (17)	C24—C210	1.4194 (17)
C14—H14	0.9500	C24—H24	0.9500
C15—C16	1.3618 (19)	C25—C26	1.3638 (19)
C15—C110	1.4170 (18)	C25—C210	1.4175 (17)
C15—H15	0.9500	C25—H25	0.9500
C16—C17	1.4059 (19)	C26—C27	1.4074 (18)
C16—H16	0.9500	C26—H26	0.9500
C17—C18	1.3700 (18)	C27—C28	1.3681 (16)
C17—H17	0.9500	C27—H27	0.9500
C18—C19	1.4219 (16)	C28—C29	1.4228 (16)
C18—H18	0.9500	C28—H28	0.9500
C19—C110	1.4239 (17)	C29—C210	1.4264 (15)
C111—C116	1.3970 (16)	C211—C216	1.3932 (17)
C111—C112	1.4003 (16)	C211—C212	1.3987 (16)
C112—C113	1.3784 (18)	C212—C213	1.3824 (16)
C112—H112	0.9500	C212—H212	0.9500
C113—C114	1.3959 (17)	C213—C214	1.3944 (17)
C113—H113	0.9500	C213—H213	0.9500
C114—C115	1.3938 (17)	C214—C215	1.3961 (17)
C114—C41	1.4400 (18)	C214—C42	1.4390 (16)
C115—C116	1.3845 (18)	C215—C216	1.3849 (16)
C115—H115	0.9500	C215—H215	0.9500
C116—H116	0.9500	C216—H216	0.9500
C41—N41	1.1477 (17)	C42—N42	1.1480 (16)
			
C12—C11—C19	119.21 (11)	C22—C21—C29	119.52 (10)
C12—C11—C111	119.27 (11)	C22—C21—C211	119.00 (10)
C19—C11—C111	121.47 (10)	C29—C21—C211	121.43 (10)
C11—C12—C13	121.72 (12)	C21—C22—C23	121.39 (11)
C11—C12—H12	119.1	C21—C22—H22	119.3
C13—C12—H12	119.1	C23—C22—H22	119.3
C14—C13—C12	120.26 (11)	C24—C23—C22	120.24 (11)
C14—C13—H13	119.9	C24—C23—H23	119.9
C12—C13—H13	119.9	C22—C23—H23	119.9
C13—C14—C110	120.38 (12)	C23—C24—C210	120.54 (11)
C13—C14—H14	119.8	C23—C24—H24	119.7
C110—C14—H14	119.8	C210—C24—H24	119.7
C16—C15—C110	121.25 (13)	C26—C25—C210	121.17 (11)
C16—C15—H15	119.4	C26—C25—H25	119.4
C110—C15—H15	119.4	C210—C25—H25	119.4
C15—C16—C17	119.98 (12)	C25—C26—C27	120.08 (11)
C15—C16—H16	120.0	C25—C26—H26	120.0
C17—C16—H16	120.0	C27—C26—H26	120.0
C18—C17—C16	120.56 (12)	C28—C27—C26	120.48 (12)
C18—C17—H17	119.7	C28—C27—H27	119.8
C16—C17—H17	119.7	C26—C27—H27	119.8
C17—C18—C19	121.00 (12)	C27—C28—C29	121.11 (11)
C17—C18—H18	119.5	C27—C28—H28	119.4
C19—C18—H18	119.5	C29—C28—H28	119.4
C18—C19—C110	118.11 (11)	C28—C29—C210	118.10 (10)
C18—C19—C11	123.17 (11)	C28—C29—C21	123.21 (10)
C110—C19—C11	118.65 (10)	C210—C29—C21	118.62 (10)
C15—C110—C14	121.12 (12)	C25—C210—C24	121.30 (11)
C15—C110—C19	119.10 (11)	C25—C210—C29	119.05 (11)
C14—C110—C19	119.76 (11)	C24—C210—C29	119.64 (11)
C116—C111—C112	118.44 (11)	C216—C211—C212	118.85 (11)
C116—C111—C11	119.89 (10)	C216—C211—C21	119.74 (10)
C112—C111—C11	121.66 (10)	C212—C211—C21	121.40 (11)
C113—C112—C111	121.08 (11)	C213—C212—C211	120.99 (11)
C113—C112—H112	119.5	C213—C212—H212	119.5
C111—C112—H112	119.5	C211—C212—H212	119.5
C112—C113—C114	119.70 (11)	C212—C213—C214	119.39 (11)
C112—C113—H113	120.1	C212—C213—H213	120.3
C114—C113—H113	120.1	C214—C213—H213	120.3
C115—C114—C113	120.15 (12)	C213—C214—C215	120.43 (11)
C115—C114—C41	120.03 (11)	C213—C214—C42	120.02 (11)
C113—C114—C41	119.82 (11)	C215—C214—C42	119.55 (11)
C116—C115—C114	119.55 (11)	C216—C215—C214	119.44 (11)
C116—C115—H115	120.2	C216—C215—H215	120.3
C114—C115—H115	120.2	C214—C215—H215	120.3
C115—C116—C111	121.07 (11)	C215—C216—C211	120.91 (11)
C115—C116—H116	119.5	C215—C216—H216	119.5
C111—C116—H116	119.5	C211—C216—H216	119.5
N41—C41—C114	179.38 (15)	N42—C42—C214	179.76 (18)
			
C19—C11—C12—C13	1.65 (17)	C29—C21—C22—C23	2.10 (19)
C111—C11—C12—C13	179.14 (10)	C211—C21—C22—C23	179.58 (12)
C11—C12—C13—C14	−1.52 (18)	C21—C22—C23—C24	−2.0 (2)
C12—C13—C14—C110	0.03 (18)	C22—C23—C24—C210	0.1 (2)
C110—C15—C16—C17	0.7 (2)	C210—C25—C26—C27	0.8 (2)
C15—C16—C17—C18	0.0 (2)	C25—C26—C27—C28	−0.1 (2)
C16—C17—C18—C19	−0.49 (19)	C26—C27—C28—C29	−0.6 (2)
C17—C18—C19—C110	0.27 (17)	C27—C28—C29—C210	0.50 (18)
C17—C18—C19—C11	−176.63 (11)	C27—C28—C29—C21	−176.45 (12)
C12—C11—C19—C18	176.55 (11)	C22—C21—C29—C28	176.52 (12)
C111—C11—C19—C18	−0.88 (17)	C211—C21—C29—C28	−0.89 (18)
C12—C11—C19—C110	−0.34 (16)	C22—C21—C29—C210	−0.41 (18)
C111—C11—C19—C110	−177.77 (10)	C211—C21—C29—C210	−177.83 (11)
C16—C15—C110—C14	177.62 (12)	C26—C25—C210—C24	177.80 (13)
C16—C15—C110—C19	−0.89 (18)	C26—C25—C210—C29	−0.9 (2)
C13—C14—C110—C15	−177.25 (11)	C23—C24—C210—C25	−177.11 (13)
C13—C14—C110—C19	1.25 (17)	C23—C24—C210—C29	1.55 (19)
C18—C19—C110—C15	0.40 (16)	C28—C29—C210—C25	0.21 (18)
C11—C19—C110—C15	177.45 (10)	C21—C29—C210—C25	177.31 (11)
C18—C19—C110—C14	−178.13 (10)	C28—C29—C210—C24	−178.48 (11)
C11—C19—C110—C14	−1.08 (16)	C21—C29—C210—C24	−1.38 (18)
C12—C11—C111—C116	−56.88 (15)	C22—C21—C211—C216	−57.00 (16)
C19—C11—C111—C116	120.55 (12)	C29—C21—C211—C216	120.43 (13)
C12—C11—C111—C112	122.22 (12)	C22—C21—C211—C212	121.53 (13)
C19—C11—C111—C112	−60.36 (15)	C29—C21—C211—C212	−61.03 (16)
C116—C111—C112—C113	−1.28 (18)	C216—C211—C212—C213	−0.71 (18)
C11—C111—C112—C113	179.61 (11)	C21—C211—C212—C213	−179.26 (11)
C111—C112—C113—C114	0.66 (18)	C211—C212—C213—C214	0.49 (18)
C112—C113—C114—C115	0.34 (18)	C212—C213—C214—C215	−0.03 (18)
C112—C113—C114—C41	−179.11 (11)	C212—C213—C214—C42	179.87 (11)
C113—C114—C115—C116	−0.69 (18)	C213—C214—C215—C216	−0.20 (18)
C41—C114—C115—C116	178.75 (11)	C42—C214—C215—C216	179.90 (11)
C114—C115—C116—C111	0.05 (18)	C214—C215—C216—C211	−0.03 (19)
C112—C111—C116—C115	0.92 (18)	C212—C211—C216—C215	0.47 (18)
C11—C111—C116—C115	−179.96 (11)	C21—C211—C216—C215	179.05 (11)

### Hydrogen-bond geometry (Å, °)

**Table:**

Cg1, Cg3, Cg5, Cg6 and Cg7 are the centroids of the C11–C110, C111–C116, C21–C210, C28–C210 and C211–C216 rings, respectively.

**Table:**

*D*—H···*A*	*D*—H	H···*A*	*D*···*A*	*D*—H···*A*
C113—H113···N42	0.95	2.58	3.4804 (17)	158
C14—H14···Cg6^i^	0.95	2.99	3.9165 (14)	165
C15—H15···Cg5^i^	0.95	2.51	3.4128 (14)	160
C17—H17···Cg3^ii^	0.95	2.86	3.6648 (15)	144
C25—H25···Cg1^iii^	0.95	2.52	3.4155 (14)	158
C27—H27···Cg7^iv^	0.95	2.91	3.7205 (15)	144
C115—H115···Cg1^v^	0.95	2.81	3.5935 (13)	141
C215—H215···Cg5^v^	0.95	2.77	3.5740 (13)	143

Symmetry codes: (i) *x*, *y*+1, *z*−1; (ii) −*x*, −*y*+2, −*z*+1; (iii) *x*+1, *y*−1, *z*+1; (iv) −*x*+1, −*y*, −*z*+2; (v) *x*−1, *y*, *z*.
